# A Narrative Review of the Most Demanding Scenarios in Basketball: Current Trends and Future Directions

**DOI:** 10.5114/jhk/170838

**Published:** 2023-09-05

**Authors:** Enrique Alonso Pérez-Chao, Rubén Portes, Miguel Ángel Gómez, Nimai Parmar, Alberto Lorenzo, Sergio L. Jiménez-Sáiz

**Affiliations:** 1 Facultad de Ciencias de la Actividad Física y del Deporte, Universidad Politécnica de Madrid, Madrid, Spain.; 2 Faculty of Sports Sciences, University Alfonso X el Sabio, Villanueva de la Cañada, Spain.; 3 Club Baloncesto Breogán de Lugo, Lugo, Spain.; 4 London Sport Institute, Faculty of Science and Technology, Middlesex University, London, UK.; 5 Sport Sciences Research Centre, Faculty of Education & Sport Sciences and Interdisciplinary Studies, Universidad Rey Juan Carlos, Fuenlabrada, Spain.

**Keywords:** peak demands, team sports, synthesis, worst-case scenarios, game demands

## Abstract

Since the analysis of most demanding scenarios (MDS) in basketball has improved the practical knowledge about match demands and possible impacts for the training process, it seems important to summarize the scientific evidence providing useful information and future directions related to MDS. This review assesses the results reflected in the available literature about the MDS in basketball, synthesizing and discussing data from scientific papers, and then providing relevant insights about terminology, sex and sample size, competition category, workload variables recorded, technology used, method of calculation, time windows analyzed, and activities evaluated related to MDS. Therefore, the present narrative review would be of practical use for coaches, scientists, athletes as well as strength and conditioning trainers exploring the current trends and future directions related to MDS in basketball.

## Introduction

Technological innovations including local positioning systems (LPSs) (Hodder et al., 2020; Serpiello et al., 2018), as well as inertial movement units (IMUs) such as accelerometers and gyroscopes (Chambers et al., 2015; Gabbett, 2013) allow to register numerous data, enabling practitioners to quantify training loads and game demands in indoor sports such as basketball. Using this information, basketball practitioners attempt to expose players to training workouts that adequately prepare them to deal with close-related demands during game-play (Alonso et al., 2020). Recently, increasingly greater attention has been paid to the most demanding periods of matches in different team sports such as basketball (Alonso et al., 2020; Fox et al., 2021a; Krolikowska et al., 2023), soccer (Novak et al., 2021; Riboli et al., 2021), futsal (Illa et al., 2020, 2021; Illa and Tarragó, 2020), rugby (Cunningham et al., 2018; Lacome et al., 2017) and hockey (Cunniffe et al., 2021; Fernández et al., 2020). In this regard, considering game demands using average values drastically underestimates the peak demands and does not take the natural intermittence of the game into account (Alonso et al., 2020; Cunningham et al., 2018). In turn, quantifying the most demanding scenarios (MDS) experienced during games is essential to tailor specific training plans that would prepare players to best endure game demands while successfully executing key technical skills (Alonso et al., 2020).

The quick growth of scientific publications means that the terminology applied varies and is used interchangeably, generating different perspectives and conflicts on the semantics of this concept. “Most demanding periods/passages”, “most intense periods”, “high intensity periods”, “worst case scenarios” and “peak demand” tend to be the most common terms that researchers used to refer to the most intense activity experienced by players for a selected variable across a specified timeframe of interest (Alonso et al., 2020). However, there exist differences regarding the terminology that need to be discussed and clarified.

Nowadays, the literature regarding MDS in basketball has analyzed the impact of contextual variables such as the moment of the game (Alonso Pérez-Chao et al., 2022a; Fox et al., 2021c), age (García et al., 2021), the type of activity (e.g., training or game) (Fox et al., 2021a), playing time (Alonso Pérez-Chao et al., 2021b; Fox et al., 2021a), the score outcome (Fox et al., 2021b), and the position or the playing role (Alonso et al., 2020; Fox et al., 2021a) that have direct or indirect influence on peak match values. Then, a wide range of different time windows (15, 30, 45 s or 1, 2, 3, 4, 5 and 10 min) and variables (e.g., distance, player load) are currently used to identify the MDS.

A previous review explored the MDS derived from IMUs on football codes (i.e., soccer, rugby union, rugby sevens, rugby league, Australian Football and Gaelic Football) (Whitehead et al., 2018). It was reported that moving averages were considered the most appropriate method for identifying peak match demands (Whitehead et al., 2018). In turn, another recent review summarized the evidence related with the MDS in soccer (Rico-González et al., 2022). However, at that time no basketball studies were included in any review. Since then, considerable information has emerged on MDS in basketball which deserves individual attention rather than inferring results from a range of ball-based team sports where the key physical and skill demands may be different. Being an approach yet to be developed and deepened, there are many features to be defined and problems to be solved. Hence, this narrative report attempts to review and discuss different aspects related to the MDS such as terminology, sex and sample size, competition category, workload variables recorded, technology used, the method of MDS calculation, time windows analyzed and activities evaluated in the study, including: (1) training and competition, (2) official competition or non-official competition only, and (3) training only. In addition, since the analysis of MDS has improved the knowledge about match demands and possible impacts for the training process, it seems important to summarize the evidence (Table 1) and discuss different considerations and future directions based on the current literature. Therefore, a comprehensive review in this area would serve coaches, scientists, athletes as well as strength and conditioning trainers in exploring current trends and future directions related to MDS in basketball.

**Table 1a:** Data summary of the articles reviewed.

Article	Term	Gender	Sample size	Competition category	External workload variables	Technology	Method calculation	Time windows	Activity
Alonso et al. (2020)	Peak Demand	Male	12 male players and 8 official games	U18 Elite players	Player Load	T6, Catapult Sports, Melbourne, Australia	Rolling average	1-min, 5-min, and 10-min	Competition
Alonso Pérez-Chao et al. (2021b)	Peak demand	Male	13 players and 9 official games	U18 Elite players	Player Load, total distance covered, distance covered in different velocity-mediated intensity zones [standing-walking (<7.0 km•h^−1^), jogging (7.0–14.0 km•h^−1^), running (14.01–18.0 km•h^−1^) and high-speed running (>18 km•h^−1^)], accelerations (>2 m•s^−2^) and decelerations (>2 m•s^−2^)	S7, Catapult Sports, Melbourne, Australia	Rolling average	30-s, 45-s, 1-min, 2-min, and 5-min	Competition
Alonso Pérez-Chao et al. (2022a)	Peak demand	Male	13 male players and 9 official games	U18 Elite players	Player Load, total distance covered, distance covered in different velocity-mediated intensity zones [standing-walking (<7.0 km•h^−1^), jogging (7.0–14.0 km•h^−1^), running (14.01– 18.0 km•h^−1^) and high-speed running (>18 km•h^−1^)], accelerations (>2 m•s^−2^) and decelerations (>2 m•s^−2^)	S7, Catapult Sports, Melbourne, Australia	Rolling average	30-s, 45-s, 1-min, 2-min, and 5-min	Competition
García et al. (2021)	Most demanding scenarios	Male	64 players and 8 games	U12, U14, U16, U18, and senior players	Total distance covered, high-speed running (>18 km•h^−1^), the number of accelerations (>2 m•s^−2^) and the number of decelerations (>2 m•s^−2)^	WIMU PRO, Realtrack Systems S.L., Almería, Spain	Rolling average	1-min	Competition
García et al. (2022)	Most demanding scenarios	Male	13 players, 17 games and 39 training weeks	Professional players	Total distance covered, distance covered in different velocity-mediated intensity zones [zone 1: stationary/walking (<6.0 km•h^−1^), zone 2: jogging (6.0–12.0 km•h^−1^), zone 3: running (12.1– 18.0 km•h^−1^), zone 4: high-intensity running (18.1–24.0 km•h^−1^), and zone 5: sprinting (>24.0 km•h^−1^)], accelerations (>2 m•s^−2^), decelerations (>2 m•s^−2^) and distance covered at accelerations (>2 m•s^−2^) and decelerations (>2 m•s^−2^)	WIMU PRO, Realtrack Systems S.L., Almería, Spain	Rolling average	1-min	Training and competition
Fox et al. (2021a)	Peak demand	Male	8 players and 15-week competitive season	Semi-professional players	Player Load	OptimEye S5, Catapult Sports, Australia	Rolling average	30-s, 1-min, 2-min, 3-min, 4-min, and 5-min	Training and competition

**Table 1b:** Data summary of the articles reviewed.

Fox et al. (2021b)	Peak demand	Male	8 players and 18 official games	Semi-professional players	Player Load	OptimEye S5, Catapult Sports, Melbourne, Australia	Rolling average	15-s, 30-s, 1-min, 2-min, 3-min, 4-min, and 5-min	Competition
Fox et al. (2021c)	Peak demand	Male	8 players and 18 official games	Semi-professional players	Player Load	OptimEye S5, Catapult Sports, Australia	Rolling average	15-s, 30-s, 1-min, 2-min, 3-min, 4-min, and 5-min	Competition
Salazar and Castellano (2019)	Most demanding passages	Male	9 players and 1 official game	Semi-professional players	Distance covered and Player Load	T6, Catapult Sports, Melbourne, Australia	Rolling average	15-s, 30-s, 45-s, 1-min, and 1-min 30-s	Competition
Vázquez-Guerrero et al. (2020)	Most demanding scenarios	Male	94 players and 13 official games	U18 Elite players	Total distance covered, distance covered in different velocity-mediated intensity zones [zone 1: stationary/walking (<6.0 km•h^−1^), zone 2: jogging (6.0–12.0 km•h^−1^), zone 3: running (12.1– 18.0 km•h^−1^), zone 4: high-intensity running (18.1–24.0 km•h^−1^), and zone 5: sprinting (>24.0 km•h^−1^)], accelerations (>2 m•s^−2^), decelerations (>2 m•s^−2^) and distance covered at accelerations (>2 m•s^−2^) and decelerations (>2 m•s^−2^)	WIMU PRO, Realtrack Systems S.L., Almería, Spain	Rolling average	10-s, 60-s, 3-min, and 5-min	Competition
Vázquez-Guerrero and García (2021)	Most demanding scenarios	Male	21 players and 1 friendly match	Professional players	Total distance covered, high-speed running (>18 km•h^−1^), the number of accelerations (>2 m•s^−2^), the number decelerations (>2 m•s^−2)^ and distance covered at accelerations (>2 m•s^−2^) and decelerations (>2 m•s^−2^)	WIMU PRO, Realtrack Systems S.L., Almería, Spain	Rolling average	1-min	Simulated competition
Vázquez-Guerrero et al. (2021)	Most demanding scenarios	Male	12 players and 10-week period	Professional players	Total distance covered, high-speed running (>18 km•h^−1^), the number of accelerations (>2 m•s^−2^) and the number decelerations (>2 m•s^−2^)	WIMU PRO, Realtrack Systems S.L., Almería, Spain	Rolling average	30-s, 1-min, and 3-min	Training and competition

### Types of Most Demanding Scenarios

Semantics deals with the linguistic meaning of words and the meaning resulting from their combination, that is, it studies the meaning of words, as well as the various meaning relationships that are established between them (Espinal et al., 2020). Therefore, in this section it is intended to define, from a pragmatic perspective, the different terminologies applied.

The MDS could be defined as multifactorial phenomena where maximal actions or actions close to the maximum intensity (over 80% of the maximum intensity) occur, in a specific period of time, considering different variables (physical, mental, environmental, and circumstantial). Then, different MDS have been explored (peak demand, very high intensity period, high intensity period and worst-case scenario) (Table 2). The peak demand (PD) is defined as the most intense activity experienced by players for a selected variable across a specified timeframe of interest (Alonso et al., 2020). The very high intensity period considers a threshold between 90–99% of the reference value of the peak demand (Illa et al., 2020), while the high intensity period considers a lower and upper limit threshold of 80–90% of the reference value of the peak demand (Illa et al., 2020). Then, the worst-case scenario should be recognized as a complex, composite construct that is actually an extreme internal response elicited via various combinations of physical and contextual factors (Novak et al., 2021).

**Table 2:** Classification of the most demanding scenarios.

Type of the most demanding scenario	Definition	Example
**Peak demand**	The peak demand is defined as the most intense activity experienced by players for a selected variable across a specified timeframe of interest.	The maximum distance a player can cover in one minute is 130 m.
**Very high intensity period**	The very high intensity period considers a threshold between 90 and 99% of the reference value of the peak demand.	If the peak demand for distance in a 1-min window is 130 m, a period of very high intensity for a period of one minute is considered when the player covers between 117 (90% of the peak demand) and 130 m.
**High intensity period**	The high intensity period considers a lower and upper limit threshold of 80–90% of the reference value of the peak demand.	If the peak demand for distance in a minute window is 130 m, a period of very high intensity for a 1-min time window is considered when the player covers between 93.6 (80% of peak demand) and 117 m.
**Worst case scenario**	The worst-case scenario should be recognized as a complex construct that is actually an extreme internal response elicited via various combinations of physical and contextual factors.	The player has played 30 min in a game away. Then, during over time, there is a situation for a 1-min window where the player covers 120 m, makes 3 jumps, receives two hits, and performs 5 decelerations and 3 accelerations

**Figure 1:**
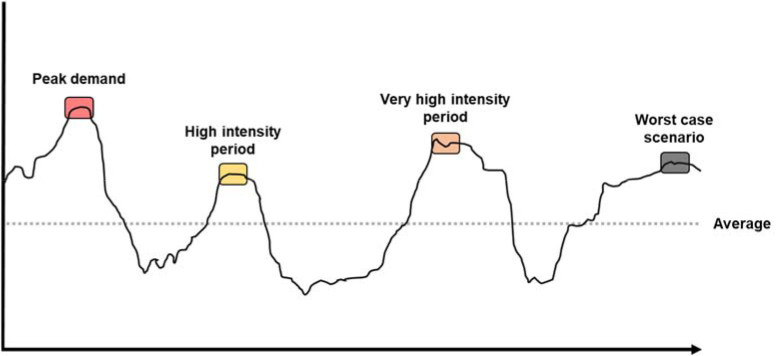
Types of the most demanding scenarios for a random variable and window duration.

Opening from the proposed classification on MDS, all publications that use the term “high intensity period” or “worst-case scenario” to refer to the most intense activity experienced by players for a selected variable across a specified timeframe of interest should use the term peak demand, in order to sustain the same mean and be able to be differentiated from the other MDS (i.e., peak demand, high intensity period or very high intensity period). The same foundation should be applied in those publications that use the term MDS or any synonym (e.g., most demanding periods or most demanding passages) to refer to peak demands. However, from a pragmatic and semantic perspective, the term MDS could also be contemplated as valid. Since a MDS can be a peak demand and a peak demand is considered a MDS, thus both terms could be used without distinction, although peak demand might be more accurate. To date, all basketball studies have been focused on peak demands, while the remainder of the MDS (high intensity period, very high intensity period and worst-case scenarios) have not been analyzed in depth. Figure 1 represents for the same window of time, the classification of MDS that can occur and should be controlled during training sessions and matches.

### Sample Characteristics

From 12 scientific papers analyzed, four of them were carried out with U18 elite male players (Alonso et al., 2020; Alonso Pérez-Chao et al., 2021b, 2022a; Vázquez-Guerrero et al., 2020), four with semiprofessional male players (Fox et al., 2021a, 2021b, 2021c), three with professional male players (García et al., 2022; Vázquez-Guerrero et al., 2020; Vázquez-Guerrero and Garcia, 2021) and one with youth and senior male elite players (García et al., 2021). No study that focused on female players was reported. In turn, three studies evaluated the peak demands during training and competition (Fox et al., 2021a; García et al., 2022; Vázquez-Guerrero et al., 2020), most studies (n = 8) (Alonso et al., 2020; Alonso Pérez-Chao et al., 2021b, 2022a; Fox et al., 2021b, 2021c; García et al., 2021; Salazar and Castellano, 2019; Vázquez-Guerrero et al., 2020) evaluated official competition only, one no official game (Vázquez-Guerrero and Garcia, 2021), while no study reported data from isolated training sessions.

### Workload Variables

The external training load is the load performed (e.g., distance), which is determined by the organization, quality, and quantity of exercise (training plan), while the internal load (e.g. heart rate) is defined as the psycho-physiological response during exercise to cope with the requirements elicited by fitness capacities and the external load applied (Impellizzeri et al., 2018). According to this rationale, no peak values for internal load variables have been analyzed, however, peak demands were calculated for a wide range of different external physical workload variables (Alonso et al., 2020; Alonso Pérez-Chao et al., 2021b, 2022a; Fox et al., 2021a, 2021b, 2021c ; García et al., 2021, 2022; Salazar and Castellano, 2019; Vázquez-Guerrero et al., 2020, 2021; Vázquez-Guerrero and Garcia, 2021), including the player’s load (Alonso et al., 2020; Alonso Pérez-Chao et al., 2021b, 2022a; Fox et al., 2021a, 2021b, 2021c; Salazar and Castellano, 2019), distance (Alonso Pérez-Chao et al., 2021b, 2022a; García et al., 2021; Salazar and Castellano, 2019; Vázquez-Guerrero et al., 2020, 2021; Vázquez-Guerrero and Garcia, 2021), high-speed distance (>18 km•h^−1^ or 18.1–24.0 km•h^−1^) (Alonso Pérez-Chao et al., 2021b, 2022a; García et al., 2021; Vázquez-Guerrero et al., 2020, 2021; Vázquez-Guerrero and Garcia, 2021), the number of actions at high intensity (> 18km•h^−1^) (Vázquez-Guerrero and Garcia, 2021), distance covered in different non-consistent velocity-mediated intensity zones [standing-walking (<7.0 km•h^−1^ or <6.0 km•h^−1^), jogging (7.0–14.0 km•h^−1^ or 6.0–12.0 km•h^−1^), running (14.01– 18.0 km•h^−1^ or 12.1–18.0 km•h^−1^) and sprinting (>24.0 km•h^−1^)] (Alonso Pérez-Chao et al., 2021b, 2022a; Vázquez-Guerrero et al., 2020), distance covered during accelerations (>2 m•s^−2^) and decelerations (>2 m•s^−2^) (Alonso Pérez-Chao et al., 2022a; Vázquez-Guerrero et al., 2020; Vázquez-Guerrero and Garcia, 2021) and the number of accelerations (>2 m• s^−2^) and decelerations (>2 m•s^−2^) (Alonso Pérez-Chao et al., 2021b, 2022a; García et al., 2021; Vázquez-Guerrero et al., 2020, 2021; Vázquez-Guerrero and Garcia, 2021).

### Methods of Calculation

The rolling averages method, which is the most accurate procedure for identifying the peak demands (Cunningham et al., 2018), has been applied in all basketball studies (Alonso et al., 2020; Alonso Pérez-Chao et al., 2021b, 2022a; Fox et al., 2021a, 2021b, 2021c; García et al., 2021, 2022; Salazar and Castellano, 2019; Vázquez-Guerrero et al., 2020, 2021; Vázquez-Guerrero and Garcia, 2021). To guarantee high quality data, it might be extracted at 1-s intervals for each player and each variable, as well as the computation of moving averages must start at the beginning of each quarter and cease at the end of the same quarter. In addition, all players should be previously familiarized with the monitoring technology as well as each player should wear the same device through the study period to avoid inter-unit variation in output (Bastida-Castillo, et al., 2018a).

### Time Duration Windows

A wide range of time windows/sample duration such as 15-s (Fox et al., 2021b, 2021c ; Salazar and Castellano, 2019; Vázquez-Guerrero et al., 2021), 30-s (Alonso Pérez-Chao et al., 2021b, 2022a; Fox et al., 2021a, 2021b, 2021c; Salazar and Castellano, 2019; Vázquez-Guerrero et al., 2020), 45-s (Alonso Pérez-Chao et al., 2021b, 2022a; Salazar and Castellano, 2019), 1-min (Alonso et al., 2020; Alonso Pérez-Chao et al., 2021b, 2022a; Fox et al., 2021a, 2021b, 2021c; García et al., 2021; Salazar and Castellano, 2019; Vázquez-Guerrero et al., 2021; Vázquez-Guerrero and Garcia, 2021), 2-min (Alonso Pérez-Chao et al., 2021b, 2022a; Fox et al., 2021a, 2021b, 2021c; Vázquez-Guerrero et al., 2020), 3-min (Fox et al., 2021a, 2021b, 2021c; Vázquez-Guerrero et al., 2021), 4-min (Fox et al., 2021a, 2021b, 2021c), 5-min (Alonso et al., 2020; Alonso Pérez-Chao et al., 2021b, 2022a; Fox et al., 2021a, 2021b, 2021c; Vázquez-Guerrero et al., 2020) and 10-min duration (Alonso et al., 2020) is currently used to identify the MDS in basketball. Analysis of these scenarios in different sample duration has several justifications since these provide different perspectives. The reason why the 1-min window is the most analyzed time frame is because it allows to compare peak demands with average demands in a simple way (Alonso et al., 2020; Salazar and Castellano, 2019). However, relativizing per minute the demands accumulated in each time window also allows to compare average demands with peak demands in different time windows, without the need for these to be the 1-min window. That is, if in 2 min a player accumulates 280 m, relativized per minute it would be 140 m covered per minute, which is easy to compare with the average demand normalized per minute.

Short windows (15, 30, 45 s and 1 min) provide information on peak values related with the nature of the sport, since generally the actions in basketball are intermittent and do not exceed the consecutive minute of duration, while long windows (2, 3, 4, 5 and 10 minutes) allow basketball practitioners to compare the peak demand with the drills, since, although it depends on the culture of each coach, the duration of these practice drills usually oscillates between 4 and 10 minutes. In fact, 30-s, 1-min, 2-min, and 5-min windows have been identified as the most practical to consider in basketball (Alonso et al., 2020; Fox et al., 2021c; Vázquez-Guerrero et al., 2020).

### Technology

Peak values are derived from technological innovations such as electronic performance and tracking systems (EPTS), including the LPS (Hodder et al., 2020; Serpiello et al., 2018), as well as IMUs such as accelerometers or gyroscopes (Chambers et al., 2015; Gabbett, 2013). The technology/manufacturer utilized through all basketball studies (Alonso et al., 2020; Alonso Pérez-Chao et al., 2021b, 2022a; Fox et al., 2021a, 2021b, 2021c; García et al., 2021, 2022; Salazar and Castellano, 2019; Vázquez-Guerrero et al., 2020, 2021; Vázquez-Guerrero and Garcia, 2021) were as follows: (I) OptimEye S5 (Fox et al., 2021a, 2021b, 2021c), Catapult Sports, Melbourne, Australia; (II) T6 (Alonso et al., 2020; Salazar and Castellano, 2019), Catapult Sports, Melbourne, Australia; (III) S7 (Alonso Pérez-Chao et al., 2021b, 2022a) Catapult Sports, Melbourne Australia; and (IV) WIMU PRO, Realtrack Systems S.L., Almería, Spain (García et al., 2021, 2022; Vázquez-Guerrero et al., 2020, 2021; Vázquez-Guerrero and Garcia, 2021).

Until now, the literature regarding the MDS in basketball has analyzed the impact of contextual-related variables such as the team level, the moment of the game (Alonso Pérez-Chao et al., 2022a; Fox et al., 2021c ), age (García et al., 2021), the type of activity (e.g., training or game) (Fox et al., 2021a), playing time (Alonso Pérez-Chao et al., 2021b; Fox et al., 2021a), the score outcome (Fox et al., 2021b), the position or the playing role (Alonso et al., 2020; Fox et al., 2021a) that have direct or indirect influence on peak match values. To date, all basketball studies have been focused on peak demands, while the remainder of the MDS (high intensity period, very high intensity period and worst-case scenarios) have not been analyzed.

### Peak Match Demands’ Fluctuations

To date, studies carried out on peak demands’ fluctuations have suggested that external peak demands decrease across the whole game (Alonso Pérez-Chao et al., 2022a; Fox et al., 2021c) for most variables analyzed. Then, peak intensities for running based demands (distance, player load and high-speed running) decrease across basketball games with most notable declines occurring between the first and fourth quarters (Alonso Pérez-Chao et al., 2022a).

Despite the scarce literature available, it seems that external peak values decrease throughout the match-play. The hypothesis that support this phenomenon may be attributed to fatigue-related mechanisms associated to playing times accumulated across entire games and prior to intense passages (Alonso Pérez-Chao et al., 2021b) or may depend on situational variables such as the team lineup used when they are active in the game due to variations in players’ capacities, team cohesion, and tactical approaches, as well as the stage of the game in which they are competing (e.g., game pace may decline during latter periods) (Alonso Pérez-Chao et al., 2021b, 2022a; Bradley and Noakes, 2013; García et al., 2020).

### Positional Differences

Considering the specific profiles of each player in the team could be an efficient strategy for the individualization of training and to prepare players for the MDS of the match, thus, several studies have been focused on peak demand differences between particular playing positions. Basketball progresses exponentially, and the categorization of players in positions offers a very reductionist perspective. Therefore, a new tendency pretends to establish the role and the function above the position, that is, the specific profile of each player needs to be understood based on their function and characteristics and not their position. This is because a player playing at the point guard position may have a totally different role and specific characteristics than another player occupying the same position.

Despite this rationale, some studies have analyzed differences of peak demands depending on particular positions. Alonso et al. (2020) observed that guards presented higher peak demands than forwards for player load in 5-min windows. Vázquez-Guerrero et al. (2020) found that pivots had lower peak demands than guards in walking distance (<7 km•h^−1^), accelerations (>2 m•s^−2^) and decelerations (>2 m•s^−2^). These results should not be extrapolated to other contexts, since the results of studies with small samples, which analyze differences between particular playing positions, are highly conditioned by the context of the team, the role, the function, and individual characteristics of each member of the sample (Alonso et al., 2020).

### Age Categories

To date, there is only one study that has evaluated the external peak demand differences across different age groups (U12, U14, U16, U18 and senior) (García et al., 2021). Differences between particular age groups for external peak demands were found, being greater for the senior group (distance traveled in a 1-min window) compared to U12. For high-speed distance (>18 km•h^−1^) significant differences were also found (1-min), as peak demands were significantly higher for U18 compared to U12. For accelerations (>2 m•s^−2^), the results were similar, reaching higher peak values in U16 compared to U12. For high-speed distance (>18 km•h^−1^) and accelerations (>2 m•s^−2^), U12 showed lower peak values. This may be due to different factors such as the duration of the match, which in the U12 category is shorter, or aspects related to the regulations, knowledge of the game or the athlete's maturation stage (García et al., 2021). It is worth mentioning that physical demands were shown to be substantially different between the five basketball age groups investigated, particularly regarding total distance covered and distance covered at high-speed (García et al., 2021).

### Playing Time Influence

There are only two studies analyzing the influence of playing time on basketball peak demands (Alonso Pérez-Chao et al., 2021b; Fox et al., 2021a). Data from recent research indirectly suggest that starting, male, semi-professional basketball players with greater playing times (33.2 ± 1.2 min) attain higher external PD (PlayerLoad^TM^) during games than bench players with lower playing times (8.7 ± 6.0 min) across time windows ranging from 30 s to 5 min (Fox et al., 2021a). However, the categorization of players into starter and bench groups in that previous research may be a limitation due to the fact that in basketball, bench players (non-starters) usually can play the same or more minutes than some players who are starters. Consequently, a factor that may have contributed to those findings is the fact that bench players were repeatedly substituted in and out of the game, therefore limiting their opportunity to undertake intense external loads and reach high PD across varied time windows. Another recent research classified players according to the minutes played and not according to their status (starter or non- starter), and observed the opposite, i.e., the lowest peak demands for players who played more minutes in total (25.0 ± 3.4 min) compared to players who participated fewer minutes (16.6 ± 2.4 min) (Alonso Pérez-Chao et al., 2021b).

Those findings can be attributed to the fact that players who played fewer minutes were less fatigued compared to those who completed more playing time. Fatigue mechanisms may be related to the loss of ability to generate force (Gibson and Noakes, 2004), and thus, lower peak demands. The explanation of fatigue is reinforced by the same study, since players who undertook less playing time prior to each PD episode reached higher peak external loads aggregated across varied time windows (Alonso Pérez-Chao et al., 2021b).

### Game Outcome

The only study to date that has analyzed the influence of peak demands found no differences in peak demands for player load (15-s, 30-s, 1-min, 2-min, 3-min, 4-min, and 5-min windows) between won and lost matches (Fox et al., 2021b). Thus, considering the scarce literature, reducing sports performance to a unique indicator (e.g., most demanding periods) should be avoided. Success (understood as having a greater number of wins) needs to be analyzed from a multidimensional approach where numerous variables interact in a non-linear and chaotic way.

### Training vs. Competition

Regarding peak demands, to date, there has been only one study (García et al., 2022) which compared external peak demands between training and competition. It was observed that most of external peak demands examined (distance covered, the number of accelerations and decelerations) were higher during official matches than in training sessions for 1-min windows (García et al., 2022). Moreover, external peak training demands along the microcycle analyzed failed to reach the maximum values obtained during official matches (García et al., 2022). A limitation of studies that attempt to compare the demands of training and competition is that the results are very dependent on the environment and methodology used. In addition, such results will not be generalizable. This happens because factors such as the coach replacement, players’ characteristics, the schedule congestion, the culture of the club and the coaching staff, objectives and the level of the team and the competition, will determine the way of training and competing of each team.

### Practical Implications

The MDS perspective offers valuable information to improve players’ preparation to withstand the specific situations, uncertainty and highest intensities encountered during the games. Depending on the context, these applications could be implemented during rehabilitation process, team practices or with unselected/fringe players (Alonso et al., 2020).

Regarding the return to sports training (RTT) and competition (return to play, RTP), one of the many criteria that could be considered is that the player may tolerate different MDS intensities (Alonso et al., 2020). Therefore, this approach provides additional information that may be useful to prepare specific progressions (Table 3), where players are exposed to different intensities before the RTT and RTP decision making process. In consequence, players may be able to withstand the average game demands, including different MDS (Alonso et al., 2020), to minimize the risk of re-injury (Reid et al., 2013).

**Table 3:** Application of the most demanding scenarios on the return to training and competition.

Phase	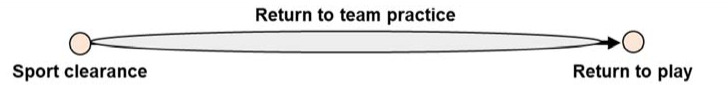		
Beginning	Medium	Final
**Scenario / Intensity**	Average demand	High intensity period Very high intensity period	Peak demand Worst case scenario
**Aim / Criteria**	Player or the injury zone should experience average intensities	Player or the injury zone should experience high and very high intensity periods	Player or the injury zone should experience peak demands and worst-case scenarios
**Control**	High	Moderate	Low
**Uncertainty / Chaos**	Low	Moderate	High

The return to competition process depends on multifactorial factors, namely the type and the injury zone. It also includes different phases where a series of criteria and objectives should be proposed (Reid et al., 2013; Taberner et al., 2019). The aim of the first phase (high control) is to increase player’s self-confidence by including in training simple movement patterns such as linear running at controlled speeds with low musculoskeletal impact (Taberner et al., 2019). The second goal of this first phase is to achieve the absence of pain during movement and to cope with running at higher intensities (Reid et al., 2013). During medium phases (moderate control), the first objective is to increase the volume and intensity of running as well as to introduce sport-specific movements (Reid et al., 2013). Athletes should be exposed to changes of direction, with or without the presence of a ball, in a controlled environment. In the final stage of RTP, it is necessary to ensure that a player is ready to cope with full team practices and deal with game demands (Reid et al., 2013). Drills should include the most realistic physiological, physical, and mental requirements to prepare players for competition (Schelling and Torres-Ronda, 2013). The goals are to expose the player to pre-injury weekly training demands and introduce drills designed to test different MDS, such as the peak demands or worst-case scenarios. In addition, some factors such as functional and radiological diagnosis, the characteristics of the injury, functional and psychological factors, player’s age, mental health, medical history, the acute chronic workload ratio and the moment of the season will affect the decision-making process regarding the final decision of returning to competitive sport (Blanch and Gabbett, 2015; Mccall et al., 2016).

Considering team practice applications, despite the scarce literature where it was observed that most of the external peak demands examined (distance covered, the number of accelerations and decelerations) were higher during official matches than in practice sessions for 1-min windows (García et al., 2022), it should be borne in mind that, in some contexts, teams have congested schedules playing 2–4 games per week, and exposing players to different MDS during training sessions may not be necessary (Alonso et al., 2020). Depending on the orientation, games per week, individual necessities, individual stress tolerance and objectives, coaches and basketball practitioners should find a way to expose players to different MDS during training sessions in order to prepare athletes to cope with the most intense demands of the game (Alonso et al., 2020). Therefore, transfer of competition demands, including both the internal and external loads, to training sessions is a difficult task. This might be attributed to factors such as the presence of fans, referees or the motivation implied by the competition itself, which is difficult to be replicated during training. Despite the characteristics of competition will hardly be replicable, in contexts with low schedule congestion (i.e., 1 match a week), players should be exposed, at least twice a week, to technical-tactical-physical requirements similar to competition, where the intermittent nature of the sport is maintained by alternating walking or jogging with different MDS (Alonso Pérez-Chao et al., 2021a).

**Figure 2:**
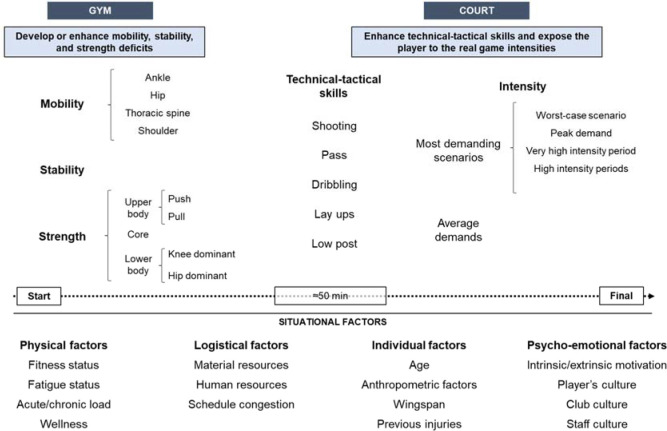
General example of a compensatory session with unselected players.

To conclude, in team sports such as basketball, the external and internal load varies greatly throughout the season between players of the same team (Manzi et al., 2010). This workload differences may be related to the presence of numerous factors that interact with each other (i.e., individual characteristics of each player, habits, role, player’s status or playing time). Such circumstances generate high variability in fitness and fatigue status within the same team. These differences between players are one of the greatest problems of team sports environments. It is evident that players with more playing time during matches accumulate more loads at the end of the week than unselected or fringe players. These differences enhance fringe or unselected players to be untrained, mainly in congested weeks with two or more games (Anderson et al., 2016; Conte et al., 2015; Gonzalez et al., 2013). Therefore, it is important to prepare particular strategies that will allow to increase the load in unselected players or those who played only few minutes (Caparrós et al., 2018; Gonzalez et al., 2013). When choosing a strategy to compensate the workload, it is essential to understand the context of each player due to the many factors (e.g., fitness and fatigue status, player wellness, material and human resources, age, position, previous injuries, team culture, club culture, motivation, schedule congestion) to account for when structuring a compensatory training session (Alonso Pérez-Chao et al., 2021a). Regarding the unselected players, when a game is played away, it is likely that a part of the squad (i.e., unselected, or injured players) does not travel to play. This situation provides a good opportunity for a compensatory session (Figure 2) (Alonso Pérez-Chao et al., 2021a). In this sense, if factors such as physical status, fatigue, fixture congestion, perceptions, and predisposition of the athlete to training require that the player needs stress which he will not be exposed to during the competition, the exposure and the dose of each stimulus should be considered as well as how this will affect the athlete (Alonso Pérez-Chao et al., 2021a). This opportunity should also be used to enhance sport-specific technical-tactical-physical skills, in addition to developing or improving capacity deficits related to performance and health (i.e., stability, strength or mobility deficit) (Alonso Pérez-Chao et al., 2021a). For players who play only few minutes during the game, the number of factors to be considered increases exponentially and sparks several questions that basketball practitioners should ask themselves. When would it be the best moment for the compensatory training session? Would it be immediately after the game, the next day, two days after the game? Or it is not necessary?? (Alonso Pérez-Chao et al., 2021a). Regarding games played away, there are logistical factors (i.e., availability of facilities, travel schedule) and physical factors (i.e., travel fatigue) that will complicate a compensatory session immediately after the game. In turn, the last trend is that players prefer to travel immediately after the match and not spend a night away from home (Calleja-Gonzalez et al., 2020). All these factors will determine the most appropriate strategy, thus understanding the context of each player as well as identifying the perceptions, expectations and predisposition of the athlete are crucial to determine what decision to make (Alonso Pérez-Chao et al., 2021a).

### Conclusions and Future Directions

Understanding game demands using average values drastically underestimates the MDS of the game (Alonso et al., 2020). MDS could be defined as a multifactorial phenomenon where maximal actions or actions close to the maximum intensity (over 80% of the maximum intensity) occur, in a specific period of time, considering different variables (physical, mental, environmental, and circumstantial). Therefore, different MDS can be explored considering for example peak demands (Alonso et al., 2020), very high intensity periods (Illa and Tarragó, 2020), high intensity periods (Illa and Tarragó, 2020) and worst-case scenarios (Novak et al., 2021)). Regarding methodological considerations, moving averages are the most appropriate method for identifying peak demands (Cunningham et al., 2018). In turn, to guarantee high-quality data, they can be extracted at 1-s intervals for each player and variable. Moreover, the computation of moving averages needs to commence at the beginning of each quarter and cease at the end of the same quarter. Most commonly analyzed time windows are (I) short windows (15, 30, 45 s and 1 min) and (II) large windows (2, 3, 4, 5 and 10 min).

While the study of MDS in basketball is growing, more research is required for a fully understanding. Up to now, studies have been focused on the impact of contextual-related variables such as the moment of the game (Fox et al., 2021c ), age (García et al., 2021), the type of activity (e.g., training or competition) (Fox et al., 2021a), playing time (Alonso Pérez-Chao et al., 2021b; Fox et al., 2021a), the score outcome (Fox et al., 2021b), the position and the playing role (Alonso et al., 2020; Fox et al., 2021a) on peak match values. More studies are warranted to extend our knowledge of the previously mentioned contextual factors. Furthermore, there are still issues that have not been addressed and require further attention:

- consistency and precision regarding the applied terminology,
- MDS in elite professional leagues as well as in female athletes and referees, since most studies have been focused on male junior players,
- non-consistent velocity intensity thresholds which make practitioners and scientists implement findings that are often controversial,
- further different scenarios, namely worst-case scenarios as well as high and very high intensity scenarios that have not been analyzed yet,
- frequency at which different MDS occur,
- context of MDS using video analysis to fully understand the circumstances around MDS occur; it is important to study technical and tactical requirements alongside the MDS data.
- MDS with regard to internal physical load variables to understand the response of the organism to these different contexts.
